# Chow fed UC Davis strain female *Lepr* fatty Zucker rats exhibit mild glucose intolerance, hypertriglyceridemia, and increased urine volume, all reduced by a Brown Norway strain chromosome 1 congenic donor region

**DOI:** 10.1371/journal.pone.0188175

**Published:** 2017-12-06

**Authors:** Craig H. Warden, Ahmed Bettaieb, Esther Min, Janis S. Fisler, Fawaz G. Haj, Judith S. Stern

**Affiliations:** 1 Departments of Pediatrics, Neurobiology Physiology and Behavior, University of California, Davis, Davis, CA, United States of America; 2 Department of Nutrition, University of Tennessee, Knoxville, TN, United States of America; 3 Department of Nutrition, University of California, Davis, Davis, CA, United States of America; 4 Internal Medicine, University of California, Davis, Davis, CA, United States of America; University of Leicester, UNITED KINGDOM

## Abstract

Our objective is to identify genes that influence the development of any phenotypes of type 2 diabetes (T2D) or kidney disease in obese animals. We use the reproductively isolated UC Davis fatty Zucker strain rat model in which the defective chromosome 4 leptin receptor (*Lepr*^*faSte/faSte*^) results in fatty obesity. We previously produced a congenic strain with the distal half of chromosome 1 from the Brown Norway strain (BN) on a Zucker (ZUC) background (BN.ZUC-*D1Rat183*–*D1Rat90*). Previously published studies in males showed that the BN congenic donor region protects from some phenotypes of renal dysfunction and T2D. We now expand our studies to include females and expand phenotyping to gene expression. We performed diabetes and kidney disease phenotyping in chow-fed females of the BN.ZUC-*D1Rat183*-*D1Rat90* congenic strain to determine the specific characteristics of the UC Davis model. Fatty *Lepr*^*faSte/faSte*^ animals of both BN and ZUC genotype in the congenic donor region had prediabetic levels of fasting blood glucose and blood glucose 2 hours after a glucose tolerance test. We observed significant congenic strain chromosome 1 genotype effects of the BN donor region in fatty females that resulted in decreased food intake, urine volume, glucose area under the curve during glucose tolerance test, plasma triglyceride levels, and urine glucose excretion per day. In fatty females, there were significant congenic strain BN genotype effects on non-fasted plasma urea nitrogen, triglyceride, and creatinine. Congenic region genotype effects were observed by quantitative PCR of mRNA from the kidney for six genes, all located in the chromosome 1 BN donor region, with potential effects on T2D or kidney function. The results are consistent with the hypothesis that the BN genotype chromosome 1 congenic region influences traits of both type 2 diabetes and kidney function in fatty UC Davis ZUC females and that there are many positional candidate genes.

## Introduction

Type 2 diabetes (T2D) and kidney disease, including end-stage renal disease (ESRD), are correlated with obesity in humans [1, 2]. Additionally, obesity increases the risk of mortality from kidney disease [3–5]. Most obese people do not develop T2D or any kidney disease, so obesity itself is not directly causal for these diseases. However, these three complex traits may share some genes influencing two or more of these diseases such that obesity may increase the risk for T2D or kidney disease in some genetically sensitive people, or these diseases may share some underlying environmental causes. An epidemiological study of approximately 900,000 patients reported that the hazard ratio for death from kidney disease was 1.59 for people with BMIs between 25 and 50. Only diabetes and liver disease have higher hazard ratios of 2.16 and 1.82, respectively [3]. However, a recent study involving patients with chronic kidney disease reported that increased BMI was correlated with decreased death rates [4]. Differences between the studies may be due to a focus on chronic kidney disease versus all kidney disease or different BMI cutoffs. A large prospective cohort study reported that proteinuria and weight were the two most potent predictors of ESRD. The effects of weight were independent of diabetes and hypertension [5].

The correlation between type 2 diabetes and kidney disease is clear, but causality is less certain. Plantinga et al [6] used data from the US 1999 through 2006 National Health and Nutrition Examination Survey, which is a representative survey of the civilian, non-institutionalized US population, to determine the prevalence of chronic kidney disease (CKD) in people with diagnosed diabetes. They reported, “Fully 39.6% of people with diagnosed and 41.7% with undiagnosed diabetes had CKD; 17.7% with prediabetes and 10.6% without diabetes had CKD.” This is consistent with the hypothesis that T2D is a risk factor for CKD, but does not prove causality since these are correlations. A recent Mendelian Randomization study used 34 T2D variants in 11,502 Chinese adults to demonstrate that a higher T2D genetic risk score was associated with a higher risk of decreased estimated glomerular filtration rate (eGFR) [7]. The investigators also reported a significant association of T2D genetic risk score and macroalbuminuria. The investigators concluded, “This analysis provides evidence for the biologically plausible causal relationship between genetically determined T2D and renal insufficiency. Additional studies are needed to validate our findings and elucidate the mechanisms behind these findings.” The investigators did not estimate what percent of eGFR impairment in T2D might be caused by the genes included in the T2D genetic risk score. We hypothesize that genes that influence type 2 diabetes in the UC Davis Zucker rat may have causal effects on urinary albumin excretion and other measures of kidney function or disease.

Causal relationships between complex diseases can be investigated in animal models. Most genes that cause obesity in mice and rats cause obesity in humans and vice versa [8]. Similarly to humans, obese mice and rats do not always develop T2D and kidney disease, even when they have a Mendelian mutation in a gene such as the leptin receptor (*Lepr*) that causes massive obesity [9]. A recent review of rodent models of diabetic nephropathy concluded, “A rodent model that strongly exhibits all … features of human diabetic nephropathy has not yet been developed” and “the currently available rodent models of diabetes can be useful in the study of diabetic nephropathy by increasing our understanding of the features of each diabetic rodent model” [10].

Zucker (fatty) rats have a spontaneous mutation in the leptin receptor (*Lepr*^*faSte*^) that causes obesity [11] and other phenotypes characteristic of metabolic syndrome [12] on a chow diet. Chow-fed males develop a mild form of T2D, have significantly elevated plasma triglycerides and develop phenotypes of mild to moderate renal disease [13]. In the UC Davis male ZUC colony, T2D related traits were primarily the presence of glucose in urine and elevated fasted glucose levels [9, 14, 15]. Renal disease markers included increases of urinary albumin excretion and urine volume with age [14]. Our previous studies have demonstrated that ovariectomy decreases and estrogen increases urinary albumin excretion in female UC Davis Zucker rats [16]. Plasma triglycerides and renal injury in females judged by histology were also increased by estrogen [16]. We previously published that ESRD was one of the causes of death in the UC Davis ZUC colony [17] and that food intake restriction improves longevity [17]. We also reported that brief periods of hyperphagia increase renal disease of female UC Davis ZUC rats as judged by histological studies and urinary albumin excretion [18]. Finally, we reported that estrogen accelerates hypertriglyceridemia and glomerular injury in female ZUC rats [19].

Other studies have shown that chow-fed fatty female Zucker strain animals develop renal disease [20], but females have been reported to be resistant to developing T2D except when fed a high-fat diet [21]. Accordingly, we now test UC Davis female ZUC strain for some phenotypes of T2D and kidney function.

T2D incidence increases with age in humans. However, the oldest animals used in the present study were 28 weeks old. We have preliminary data suggesting that ad-lib chow-fed male UC Davis ZUC animals begin to spontaneously die at 28 weeks of age, so we have restricted studies of females to a maximum age of 24–28 weeks.

Congenic strains are made by breeding a donor strain to a background strain and using repeated backcrosses to the background strain to isolate a selected chromosomal region from the donor strain. Thus, congenic strains are almost identical to background strains. Phenotype differences between congenic and background are due to donor strain alleles in the selected chromosomal region. We previously reported the development of a congenic strain that has Brown Norway (BN) alleles on the distal half of chromosome 1 on a UC Davis ZUC background [14]. The formal name for the congenic is BN.ZUC-*D1Rat183*-*D1Rat90* where *D1Rat183* and *D1Rat90* are microsatellite markers polymorphic between the UC Davis ZUC and the Brown Norway rat strain. The congenic strain will be referred to as BN congenic throughout the rest of this paper [14, 15]. Thus, BN congenic fatty animals are homozygous for *Lepr*^*faSte*^ and have the BN donor region from *D1Rat183*-*D1Rat90* on a ZUC background. We previously demonstrated that the BN chromosome 1 congenic donor region decreased both T2D and renal disease in chow-fed fatty males compared to ZUC background [14]. These data are consistent with the donor region influencing T2D and renal disease.

Many genes influence the risk of developing complex diseases. When whole genome mapping data is available, strains that are sensitive or resistant to complex diseases, such as obesity, diabetes and kidney diseases often appear to be blends of genes promoting susceptibility and resistance to these complex diseases [9, 22–26]. There are hundreds of genes in the BN congenic strain donor region. Previous genetic mapping studies showed at least two separate regions on chromosome 1 that influence T2D and kidney disease [14]. Genes for susceptibility to renal disease and T2D in the BN congenic may be one and the same, or they may be different. Furthermore, quantitative trait loci (QTL) peaks, like the ones identified in the BN congenic strain, may include two or more causal genes [22, 26]. Thus, the total number of disease causal genes in the BN donor region is unknown but likely includes several or more.

We used quantitative PCR to examine the relationship of six genes with T2D and kidney disease. These six genes are located in the BN donor region of chromosome 1 but are a small subset, not a comprehensive collection, of all plausible candidate genes in the BN donor region. However, each of these six was tested in depth in the female, *Lepr* lean and fatty, and chromosome 1 congenic strain genotypes of BN and ZUC. We also examined three different parts of the kidney. The six genes examined were (1) uromodulin (*Umod*), (2) sortilin-related VPS10 domain containing receptor 1 (*Sorcs1*), (3) rab11 family-interacting protein 2 (*Rab11fip2*), (4) sodium channel, nonvoltage-gated 1, beta subunit (*Scnn1b*), (5) protein disulfide-isomerase-like protein of the testis (*Pdilt*), and (6) polycystic kidney disease 2-like 1 protein (*Pkd2l1*). We also examined the expression of these genes in three different regions of the kidney.

Missense mutations of *Umod* were previously shown to cause kidney disease, including renal failure in humans [27, 28]. Variants of *Sorcs1* were previously shown to cause T2D and to influence urinary albumin excretion in rats and humans [29, 30]. *Sorcs1* is located under a urinary albumin excretion (UAE) peak in the BN congenic at 277412523 MBp [15]. *Rab11fip2* is located at 288422015 MBp on rat chromosome 1, which is the QTL peak for UAE [15]. It was reported that *Rab11fip2* influences transport of vesicles from the endosomal recycling compartment (ERC) to the plasma membrane and is involved in the regulation of water balance by aquaporins [31, 32]. *Pkd2l1* is a paralog of *Pkd2*, which causes autosomal dominant polycystic kidney disease [33]. *Pkd2l1* has been identified as a positional candidate gene for T2D in the Goto-Kakizaki (GK) and Wistar rat models [34]. It is located at 271367373 MBp on rat chromosome 1 under the glucose QTL peak [15]. *Scnn1b* is located in a UAE QTL peak [15]. *Scnn1b* was considered to be one of the candidate genes to influence kidney function because mutations of *Scnn1b* are a cause of Liddle’s syndrome, which results in increased sodium and water reabsorption in the distal nephron *[35, 36]. Pdilt* is located at 196149077 under the QTL peaks for UAE and liver weight, adjacent to *Umod*. Genome-wide association studies in humans have found that significant effects of SNPs in both *Umod* and *Pdilt* on Umod protein levels and eGFR [37].

Our objective is not to perform comprehensive phenotyping for T2D and kidney disease, but rather to identify phenotypes in young animals that are influenced by congenic region BN genotype that can be used for genetic mapping of the underlying causal genes. Phenotypes apparent in younger animals will reduce the time needed for mapping, but carry the risk that they may or may not predict disease in older animals. Since we have substantial published data on phenotypes of young and old UC Davis ZUC strain animals and have observed correlations between ages [16–19, 38], our hypothesis is that comprehensive phenotyping of genetically modified Zucker rats produced for future work will be the final determinant of whether or not we have identified genes for obesity induced disease risk.

Our prior data is consistent with the hypothesis that chow fed fatty male UC Davis ZUC strain animals are a model for type 2 diabetes [9, 14, 15], whereas data in this manuscript is consistent with the hypothesis that fatty female UC Davis ZUC strain animals are a model of prediabetes and that the BN donor region of a chromosome 1 congenic strain reduces blood glucose levels during a glucose tolerance test, glucose excreted per day in urine from non-fasted animals, and non-fasted animal plasma triglyceride levels and urine volume.

## Methods

### Ethics statement

All protocols followed the guidelines of the American Association for Accreditation of Laboratory Animal Care (approval number 000029) and were approved by the Institutional Animal Care and Use Committee (IACUC) of the University of California, Davis (Davis, CA).

### Animals

Zucker (ZUC) fatty rats are homozygous for the leptin receptor (*Lepr*^*faSte/*faSte^) alleles that produce severe obesity. *Lepr* genotype may be either lean *Lepr*^*+Ste/+Ste*^ or fatty *Lepr*^*faSte/*faSte^. BN congenic genotype rats are homozygous for Brown Norway (BN) rat strain alleles in the BN.ZUC-*D1Rat*-*D1Rat90* congenic strain, where BN is the donor strain and ZUC is the background strain. Thus, fatty congenic means homozygous for *leptin receptor Lepr*^*faSte/faSte*^ alleles and also homozygous for BN strain alleles in the BN chromosome 1 congenic region. No heterozygote data are reported for either *Lepr* genotype or BN congenic donor region genotype.

ZUC background and BN congenic rats, lean and fatty, were bred in our UC Davis breeding colony. We previously named these as Zucker fatty rats (ZUC- *Lepr*^*faSte*^ (RGD ID: 629462)) [15], but for this paper, we propose the term UC Davis ZUC to indicate better the unique origin of the animals studied in this work. The UC Davis ZUC colony has been reproductively isolated from all other ZUC strain animals since at least 1982 [39]. For at least 10 consecutive generations the colony was maintained by breeding one brother-sister pair per generation. Although we have not assessed inbreeding by genotyping, the UC Davis ZUC colony likely has a unique genotype and may have phenotypes distinct from other ZUC strain animals. Thus, we need to measure all phenotypes directly in the UC Davis colony, since they cannot be predicted from studies in other ZUC strain animals. The BN.ZUC-*D1Rat183*-*D1Rat90* congenic strain (also abbreviated as congenic or BN congenic) was bred by repeated backcross of ZUC x BN F1s to UC Davis ZUC strain, with selection for chromosome 1 BN alleles. The *Lepr* fatty mutation was maintained as heterozygous during breeding so that we could readily generate animals that are homozygous for the *Lepr* fatty mutation for phenotyping studies. Characterization of this congenic strain in males has been published [14].

Congenic animals for phenotyping were constructed by breeding. The congenic colony is homozygous BN from *D1Rat83* to *D1Rat90* and is heterozygous for *Lepr*^*faSte/+Ste*^. Intercrosses of these animals produce progeny where 25% are wildtype lean *Lepr*^*+Ste/+Ste*^ and 25% are homozygous fatty *Lepr*^*faSte/faSte*^. Retention of BN alleles from *D1Rat183* to *D1Rat90* is confirmed for every animal phenotyped and used for breeding every generation. *Lepr*^*faSte/Ste+*^ heterozygotes produced in these same crosses were used for breeding. The ZUC colony is also maintained as *Lepr*^*faSte/+Ste*^ heterozygotes, with animals for phenotyping produced by intercross and identification of animals homozygous lean or homozygous fatty by *Lepr*^*faSte*^ versus *Lepr*^*+Ste*^ specific genotype. The fatty mutation of Zucker rats introduces an Msp1 restriction site into the *Lepr* gene. Thus the two genotypes are detected by PCR of genomic DNA surrounding the fatty mutation followed by Msp1 restriction digestion [40].

Animals were housed at the UC Davis animal facility which was maintained at a temperature between 70–72°F, a relative humidity of 50–60%, and a14/10-h light/dark cycle. Animals were fed *ad libitum* PMI LabDiet #5008 Formulab Diet and deionized water. All animals included in this study were virgins.

To measure food intake rats were single-housed on wire bottom cages and allowed to acclimate for 5–7 days. Body weight, food in, food out, and spill were measured for 48 hours for each time-point.

For urine collection, rats were weighed then housed in metabolic wire caging with funnels for 24 hours. Food and water were provided *ad libitum*. To minimize potential food particle contamination of the urine sample, food was prepared as a wet mash using a ground meal version of their normal chow. The collection funnel was fitted with a wire mesh filter and glass wool placed in the funnel tip to further minimize contamination from food particles and feces. Three drops of a 10% sodium azide solution were added to each collection beaker to inhibit bacterial growth in the urine sample. After 24 hours, urine was collected, volume measured, centrifuged, and aliquoted and frozen at -80°C until further analysis.

Prior to sacrifice animals were anesthetized with isoflurane. Blood was removed via cardiac puncture. The animal was then euthanized by an overdose of isoflurane, exsanguination, and by cutting of the diaphragm. Carcass weight was determined based on the total carcass remaining after removal of the liver, kidneys, and the gonadal, mesenteric, peritoneal, and brown adipose tissue fat pads. The organs were rapidly removed and weighed.

### Assessment of diabetes

For glucose tolerance tests (GTT), the rats were fasted overnight (12–15 hours) at 14 and 27 weeks of age. Animals were gavaged with 2 mg/g body weight of 50% D-glucose to initiate GTT. Blood glucose levels were measured at 0, 2, 15, 30, 60, and 120 minutes after injection using OneTouch Ultra Meter. The area under the curve (AUC) was calculated for plasma glucose levels during GTT using the trapezoidal method [41].

Insulin tolerance tests (ITT) were conducted at 13 and 26 weeks of age. Rats were fasted for 4 hours and then given an intraperitoneal injection of 0.75U/kg insulin (Novolin-R). Blood glucose levels were measured at 0, 15, 30, 45, 60, and 120 minutes using OneTouch Ultra Blood Glucose Meter.

### Renal function assessment

Urine albumin concentration (UAE mg/24 hr) was determined using the albumin blue 580 method [42, 43]. Urine creatinine (mg/24 hr) was measured using Assay Designs (Ann Arbor, MI) colorimetric detection kit (Cat. # 907–030). 24-hour urine volume was used to calculate the 24-hour urinary creatinine and albumin excretion. Albumin to creatinine ratio was determined as mg albumin/mg creatinine using the 24-hour values. Urine glucose was measured by enzymatic methods (hexokinase).

### Plasma assays

Non-fasted plasma samples were collected in K_3_EDTA coated tubes and sent to the Comparative Pathology Laboratory on the UC Davis Campus for assays that were performed using the Roche Diagnostics Cobas Integra 400 Plus chemistry analyzer. Briefly, the assays are based on the following methods: albumin (Bromcresol Green Indicator, Dye Binding); urea nitrogen (Urease enzymatic); triglycerides (TG) (glycerol blanked enzymatic); creatinine (enzymatic); glucose (hexokinase); total cholesterol is enzymatically measured using cholesterol esterase and cholesterol oxidase; total protein is measured using a colorimetric reaction involving the reaction of divalent copper ions in alkaline medium, to form the well-characterized purple Biuret complex with protein where color intensity is directly proportional to the protein concentration. Total protein values were used to calculate globulin levels (total protein–plasma albumin). Globulin levels were then used to calculate albumin/globulin ratios.

### Quantitative PCR

At sacrifice samples of the whole kidney, kidney cortex and kidney medulla were frozen at -80C until extraction. We took the entire medulla without the border near cortex and cortex without the border near the medulla.

RNA was extracted from the whole kidney, kidney cortex or kidney medulla samples using TRIzol (Invitrogen), and cDNA generated using high-capacity cDNA synthesis kit (Applied Biosystems). mRNA levels were assessed by SYBR Green quantitative real-time PCR using SsoAdvanced™ Universal SYBR® Green Supermix (CFX96 Touch™ Real-Time PCR Detection System, BioRad). Relative gene expression was quantitated using the 2(-Delta Delta C(T)) method with appropriate primers (Table 1) and normalized to the TATA-box binding protein (*Tbp*) as previously described [44].

**Table 1:** Primers and chromosomal locations used to quantitate *Pdilt*, *Pkd2l1*, *Rab11fip2*, *Scnn1b*, *Sorcs1*, *Tbp*, and *Umod* expressions.

Genes	Forward 5’->3’	Reverse 5'->3'	Position on rat chromosome 1
*Tbp*	TATAATCCCAAGCGGTTTGC	CAGCCTTATGGGGAACTTCA	57,491,643
*Umod*	AATTCCGGGCAGAGCACAAA	TCCGTGTTGACACAGGTAGC	189,186,026
*Pdilt*	TGCCAGGTACAAAATGCCCA	GTACTGTTCTGGGTTCGCCA	189,207,165
*Scnn1b*	TCTAGCTCTTGCCCACCCTA	CTGCACACCCCCAAGTCTAT	191,704,311
*Pkd2l1*	CGTGTCTCCTGTGAGAGTGG	TGCCCCTGTCTAGCCATAGT	263,922,201
*Sorcs1*	AGCTTTAACCTCCCCTCCCT	GTACACTGCCCCTCCAAACA	269,973,351
*Rab11fip2*	TGCAACACTGCCAAGGAAGA	TTCGTAGGTCAGACTCCGGT	281,065,168

All genes are located on rat chromosome 1. Chromosomal positions are from Ensembl Rnor_6.0:CM000072.5. *Tbp* is not inside the BN congenic donor region of the BN.ZUC-*D1Rat183*-*D1Rat90* congenic. All other genes in Table 1 are inside the BN donor region.

*Tbp* is a central eukaryotic transcription factor used by all three cellular RNA polymerases and is widely used as a housekeeping gene. Some publications comparing different control genes (including *glyceraldehyde 3-phosphate dehydrogenase*, *actin*, *ribosomal protein large P0*, and *ribosomal protein S18* in different human and rodent tissues and under different disease states indicate that *Tbp* is perhaps the most appropriate housekeeping gene for normalization of transcript abundance measured by real-time RT-PCR. In all these studies, *Tbp* exhibited the highest expression stability and low coefficient of variation values [45–51]. We have previously published use of *Tbp* as a normalizing gene for quantitative PCR [52].

### Statistical analysis

ANOVAs were calculated with JMP13 Pro. P-values are adjusted for the False Discovery Rate (FDR) [53]. Repeated measures ANOVAs were also performed using JMP13 Pro using a mixed model with age in weeks, *Lepr* genotype (fatty vs. lean) and congenic donor genotype (BN vs. ZUC) as the three fixed effects, animal number as a random effect, and repeated covariance structure of residual. Using AR(1) for repeated covariance structure did not affect on the results.

## Results

### Genotype effects on body and organ weights in females

We first compared *Lepr* genotype and BN congenic genotype effects on body and tissue weights in females at 15 and 28 weeks (Table 2). Congenic genotype has a significant effect on body weight (p<0.0002). Accordingly, we searched for congenic effects on dissected organ weights using the weights normalized to body weight, i.e., percent tissue weight. Congenic genotype has a significant effect on the weight of each organ except brown adipose tissue (BAT). *Lepr* genotype has a significant effect on all body and organ weights. However, there are no significant interactions between *Lepr* and BN genotype for body weight or the weight of any organ except MWAT. *Lepr* genotype, BN genotype, age and the interaction of age and *Lepr* genotype influence body weight and the weights of liver, kidney and several fat depots.

**Table 2:** Body weight and organ weights as a percent of body weight in Zucker lean *Lepr*^*+Ste/+Ste*^ and fatty *Lepr*^*faSte/faSte*^ female rats with either Brown Norway (BN) or Zucker (ZUC) chromosome 1 congenic donor region at 15 or 28 weeks of age. Data are mean ± SE.

Age	15 weeks				28 weeks			
*Lepr* genotype	*Lepr*^*+Ste/+Ste*^		*Lepr*^*faSte/faSte*^		*Lepr*^*+Ste/+Ste*^		*Lepr*^*faSte/faSte*^	
BN congenic genotype	BN	ZUC	BN	ZUC	BN	ZUC	BN	ZUC
Number	11	13	11	12	11	12	12	11
Body weight (g)	211 ± 4^F^	219 ± 3^EF^	381 ± 6^C^	398 ± 11^C^	233 ± 3^DE^	252 ± 2^D^	448 ± 9^B^	485 ± 13^A^
Carcass weight (g)	195 ± 3^F^	201 ± 3^EF^	337 ± 5^C^	348 ± 9^C^	216 ±3^DE^	231 ± 2^D^	398 ± 8^B^	418 ± 12^A^
Body length (cm)	20.23 ± 0.08^F^	20.70 ± 0.09^E^	22.09 ± 0.09^B^	22.13 ± 0.23^B^	21.18 ± 0.10^D^	21.63 ± 0.11^C^	22.79 ± 0.10^A^	23.14 ± 0.20^A^
Liver (%BW)	2.50 ± 0.05 ^BC^	2.62 ± 0.04 ^BC^ *	2.58 ± 0.05 ^BC^	2.75 ± 0.04 ^AB^	2.16 ± 0.02 ^D^	2.42 ± 0.02 ^CD^	2.59 ± 0.04 ^BC^	2.98 ± 0.14 ^A^
Right GWAT (%BW)	0.19 ± 0.01 ^E^	0.34 ± 0.02 ^DE^	1.95 ± 0.09 ^B^	2.22 ± 0.06 ^A^	0.21 ± 0.02 ^E^	0.45 ± 0.02 ^D^	1.68 ± 0.06 ^C^	1.97 ± 0.07 ^B^
Right RWAT (%BW)	0.08 ± 0.00 ^D^	0.14 ± 0.00 ^D^	0.90 ± 0.04 ^C^	0.91 ± 0.02 ^C^	0.08 ± 0.00 ^D^	0.20 ± 0.00 ^D^	1.25 ± 0.04 ^B^	1.58 ± 0.10 ^A^
MWAT (%BW)	0.59 ± 0.03 ^D^	0.71 ± 0.03 ^D^	2.83 ± 0.10 ^B^	3.50 ± 0.15 ^A^	0.58 ± 0.04 ^D^	1.09 ± 0.04 ^C^	2.58 ± 0.08 ^B^	3.27 ± 0.13 ^A^
BAT (%BW)	0.09 ± 0.00 ^B^	0.10 ± 0.00 ^B^	0.38 ± 0.01 ^A^	0.41 ± 0.01 ^A^	0.10 ± 0.00 ^B^	1.24 ± 0.00 ^B^	3.97 ± 0.01 ^A^	0.42 ± 0.03 ^A^
Gastrocnemius (%BW)	0.71 ± 0.01 ^A^	0.66 ± 0.00 ^B^	0.38 ± 0.00 ^D^	0.34 ± 0.00 ^E^	0.70 ± 0.00 ^A^	0.62 ± 0.00 ^C^	0.37 ± 0.01 ^D^	0.32 ± 0.00 ^E^
Left kidney (%BW)	0.66 ± 0.04 ^D^ **	0.74 ± 0.03 ^D^	1.05 ± 0.04 ^C^	1.18 ± 0.03 ^BC^	0.68 ± 0.04 ^D^ ***	0.81 ± 0.03 ^D^	1.26 ± 0.03 ^B^	1.56 ± 0.04 ^A^
Three-way ANOVA effect								
	*Lepr* genotype		BN congenic genotype	Age	*Lepr* genotype x BN congenic	*Lepr* genotype x Age		BN congenic x Age
Body weight	**<0.0001**		**0.0002**	**<0.0001**	0.2336	**<0.0001**		0.1457
Carcass weight	**<0.0001**		0.0059	**<0.0001**	0.6255	**<0.0001**		0.3756
Body length	**<0.0001**		**0.0012**	**<0.0001**	0.1680	0.6658		0.4689
Liver	**<0.0001**		**<0.0001**	0.0842	0. 2670	**<0.0001**		0.0375
Right GWAT	**<0.0001**		**<0.0001**	0.0065	0.2279	**<0.0001**		0.4253
Right RWAT	**<0.0001**		**<0.0001**	**<0.0001**	0.1883	**<0.0001**		**0.0009**
MWAT	**<0.0001**		**<0.0001**	0.6469	**0.0032**	**0.0008**		0.0966
BAT	**<0.0001**		0.0254	0.1888	0.7373	0.5537		0.8531
Gastrocnemius	**<0.0001**		**<0.0001**	**0.0002**	0.0144	0.5298		0.0094
Left kidney	**<0.0001**		**<0.0001**	**<0.0001**	0.0331	**<0.0001**		0.0326

GWAT = gonadal adipose tissue. RWAT = retroperitoneal white adipose tissue. MWAT = mesenteric white adipose tissue. BAT = brown adipose tissue. Data analysis is by three-way ANOVA (Expected Means Squares approach) with mean comparisons by Tukeys HSD: means not sharing superscript (^A, B, C, D, E, F^) are different at p≤0.05. P values in bold are significant at p = 0.05 when corrected for multiple testing using the FDR.

* N = 12

** N = 10

*** N = 9.

### Food intake

**Fig 1:**
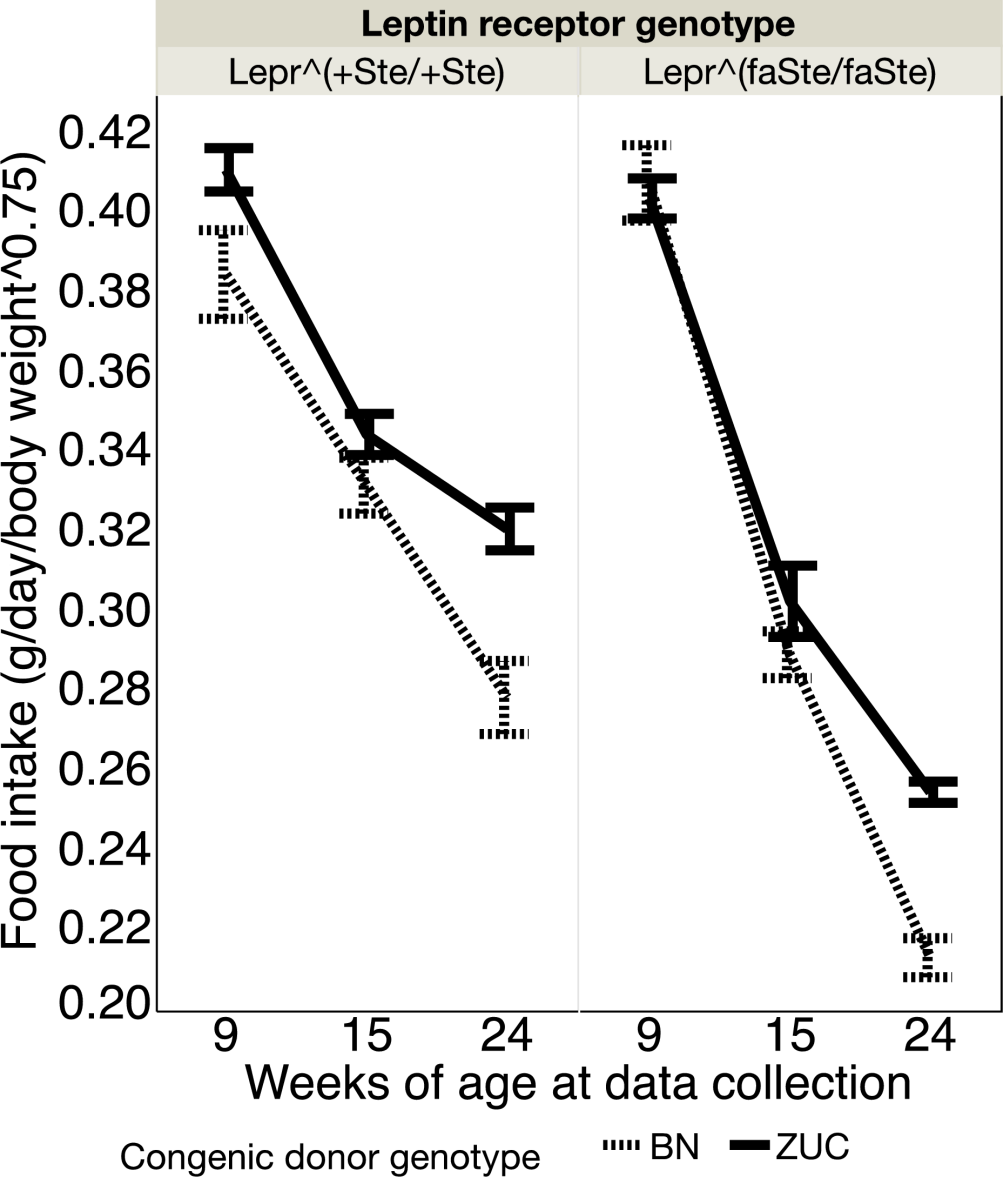
Food intake (mean ± SE, g/day/body weight^0.75^) in lean and fatty females at 9, 15 and 24 weeks of age normalized to contemporaneous measurements of body weight showing the effect of congenic genotype on food intake. Number of rats in each group: *Lepr*^*+Ste/+Ste*^, BN congenic donor genotype N = 8, ZUC congenic donor genotype N = 16; *Lepr*^*faSte/faSte*^, BN congenic donor genotype N = 10, ZUC congenic donor genotype N = 18.

Food intake per day is reported in Fig 1. Food intake was measured at 9, 15 and 24 weeks and normalized to body weight^0.75^ as this normalizes metabolic needs across varying weights. Repeated measures ANOVA showed highly significant effects of age (p<0.0001), the interaction of age with *Lepr* genotype (p<0.0001), the interaction of age with BN congenic genotype (p = 0.0055), and lesser significance for *Lepr* genotype and BN congenic genotype interaction (p = 0.0330). ZUC strain animals ate more food at 24 weeks than BN genotype congenic strain animals.

### Urine kidney function phenotypes

**Fig 2:**
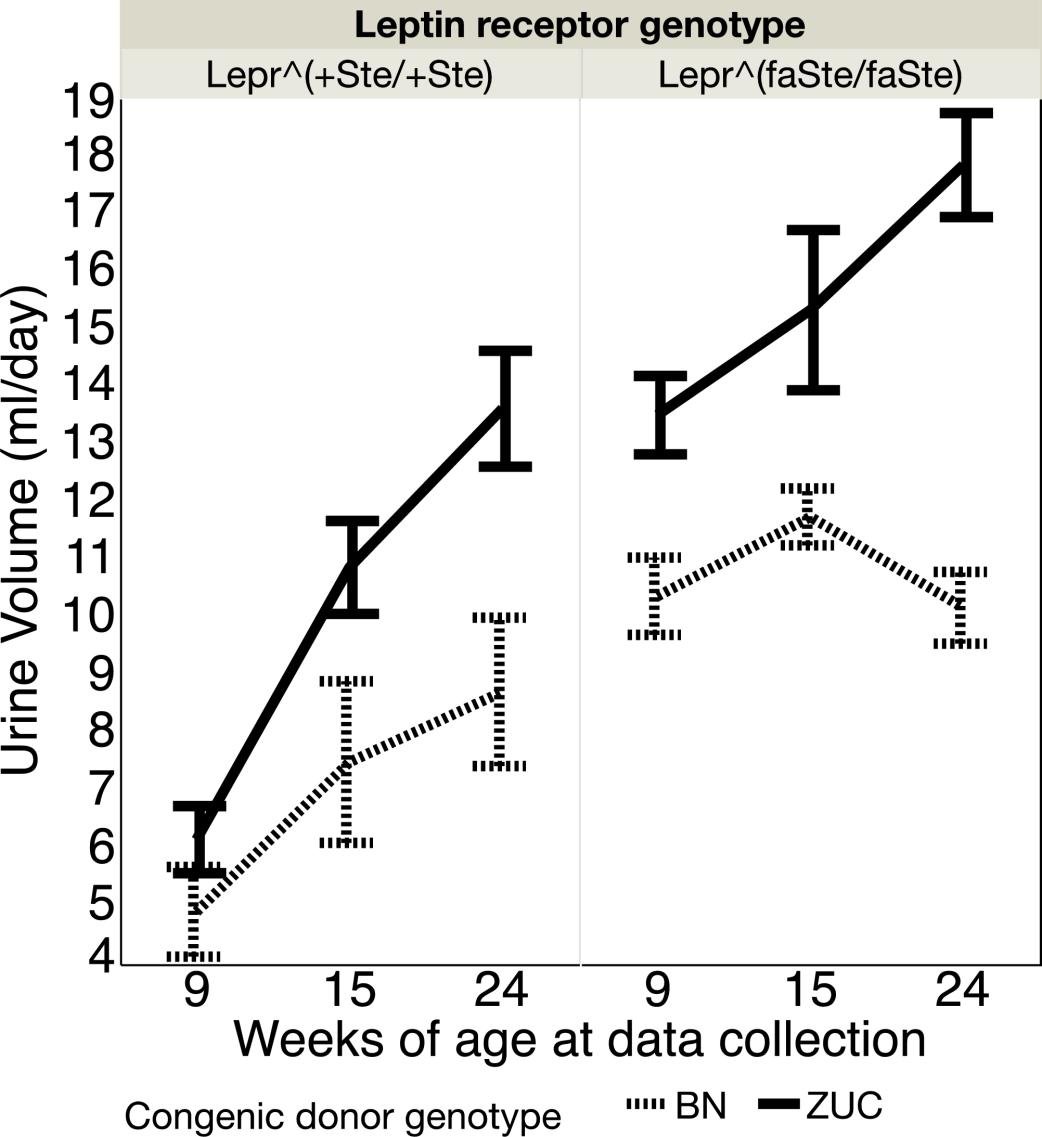
Urine volume (ml/day, mean ± SE) by age, congenic region genotype and *Lepr* genotype in female rats. Number of rats in each group: *Lepr*^*+Ste/+Ste*^, BN congenic N = 8, ZUC N = 16; *Lepr*^*faSte/faSte*^, BN congenic N = 10, ZUC N = 18.

Urine volume was also measured at 9, 15 and 24 weeks (Fig 2). Repeated measures ANOVA revealed highly significant effects of *Lepr* genotype (p<0.0001), age (p<0.0001), age interaction with congenic region genotype (p = 0.0020) and *Lepr* genotype (p = 0.0097) and a lesser effect for congenic region genotype (p = 0.0357).

**Fig 3:**
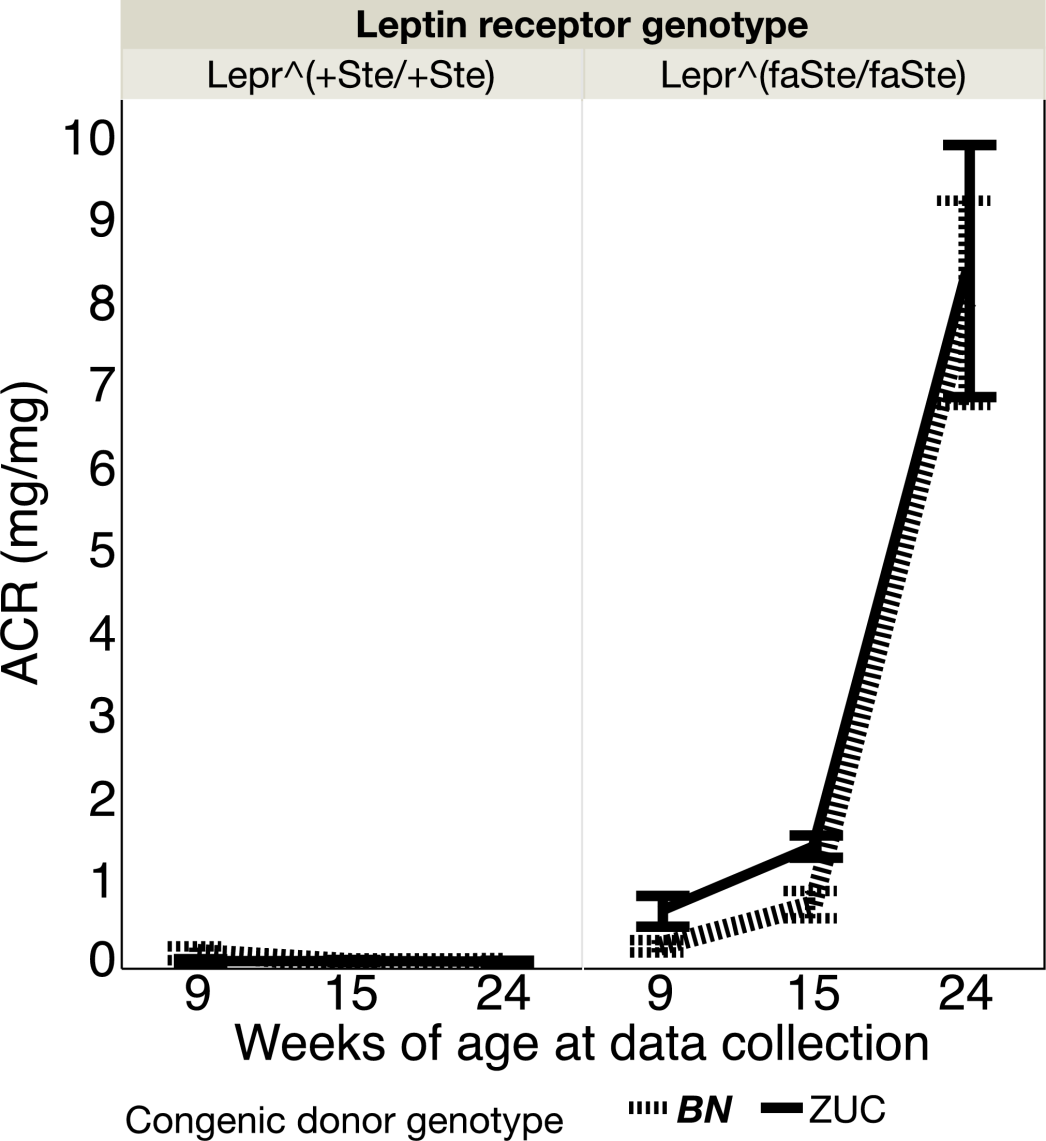
Albumin/Creatinine (ACR) (mean ± SE, ml/day) by age, congenic region genotype and *Lepr* genotype in female rats. Number of rats in each group: *Lepr*^*+Ste/+Ste*^, BN congenic N = 7, ZUC N = 6; *Lepr*^*faSte/faSte*^, BN congenic N = 10, ZUC N = 5.

Urinary albumin to urinary creatinine (ACR) ratio was also measured at 9, 15 and 24 weeks (Fig 3). Repeated measures ANOVA revealed highly significant effects of age (p<0.0001), interaction of age and *Lepr* genotype (p<0.0001), but no other statistically significant effects. For 15 week females corresponding correlations for UAE and creatinine are r^2^ = 0.25 and 0.03 for positive correlations (p<0.0002 and 0.02, n = 64 and 15, respectively). Fatty females exhibit strong age-dependent increases of ACR that are consistent with the development of kidney disease.

### Plasma phenotypes

We quantified levels of six plasma phenotypes in non-fasted 28-week old females with *Lepr*^*+Ste/+Ste*^ or *Lepr*^*faSte/faSte*^ genotypes and BN or ZUC congenic donor region genotypes. We also used these data to calculate the ratio of albumin to globulin, which is a measure of kidney function. Mean values for albumin to globulin, urea nitrogen, creatinine, glucose, total cholesterol, and triglyceride are shown in Table 3.

**Table 3:** Non-fasted plasma phenotypes in 28-week old Zucker lean *Lepr*^*+Ste/+Ste*^ and fatty *Lepr*^*faSte/faSte*^ female rats with either Brown Norway (BN) or Zucker (ZUC) chromosome 1 congenic donor region. Data are means ± SE.

*Lepr* genotype	*Lepr*^*+Ste/+Ste*^		*Lepr*^*faSte/faSte*^	
BN congenic genotype	BN	ZUC	BN	ZUC
Number	9	7	10	5
Albumin to Globulin Ratio	2.90 ± 0.06^A^	2.84 ± 0.05^A^	2.14 ± 0.10^B^	1.84 ± 0.07^B^
Creatinine (mg/dL)	0.41 ± 0.01^A^	0.34 ± 0.01^B^	0.27 ± 0.01^C^	0.20 ± 0.01^D^
Urea Nitrogen (mg/dL)	21.2 ± 0.7^A^	18.3 ± 1.0^B^	9.9 ± 0.3^C^	7.6 ± 0.5^C^
Glucose (mg/dL)	116 ± 4^B^	124 ± 3^B^	149 ± 6^A^	147 ± 5^A^
Cholesterol (mg/dL)	58 ± 3^C^	67 ± 2^BC^	89 ± 10^A^	115 ± 10^A^
Triglyceride (mg/dL)	7.2 ± 1.3^C^	14.3 ± 1.6^C^	427 ± 80^B^	937 ± 122^A^
Two-Way ANOVA				
	Effect of *Lepr* genotype	Effect of congenic genotype		Interaction
Albumin to Globulin Ratio	**<0.0001**	0.0351		0.1639
Creatinine	**<0.0001**	**<0.0001**		0.7202
Urea Nitrogen	**<0.0001**	**0.0008**		0.7155
Glucose	**<0.0001**	0.5761		0.3488
Cholesterol	**<0.0001**	0.0404		0.2833
Triglyceride	**<0.0001**	**0.0007**		**0.0009**

Data analysis is by two-way ANOVA with mean differences calculated by Tukey HSD: means not sharing a superscript (^A, B, C^) are significantly different at p = 0.05. P values in bold are significant at p = 0.05 when corrected for multiple testing using the FDR.

There were highly significant *Lepr* genotype effects for all six phenotypes and for BN congenic genotype effects for three traits, creatinine, blood urea nitrogen, and triglyceride. Tukey’s post hoc tests revealed that all four groups differ for creatinine, with fatty ZUC lowest and BN genotype lean highest. Tukey’s post hoc tests for blood urea nitrogen reveal that both congenic genotypes of fatty animals are lower than both congenic genotypes of lean. Fatty ZUC have almost twice as much triglyceride as fatty BN congenic genotype females, while lean animals have tens of fold lower levels of triglyceride than either congenic genotype fatty. Both congenic genotype fatty animals have triglyceride levels consistent with hypertriglyceridemia. Plasma glucose was higher in fatty animals than lean, with both groups of congenic genotype fatty animals having non-fasted glucose levels greater than 140 mg/dL.

### Diabetes phenotypes

We surveyed glucose homeostasis using several different assays; non-fasted plasma glucose (shown in Table 3), urine glucose, glucose tolerance tests and insulin tolerance tests.

**Fig 4:**
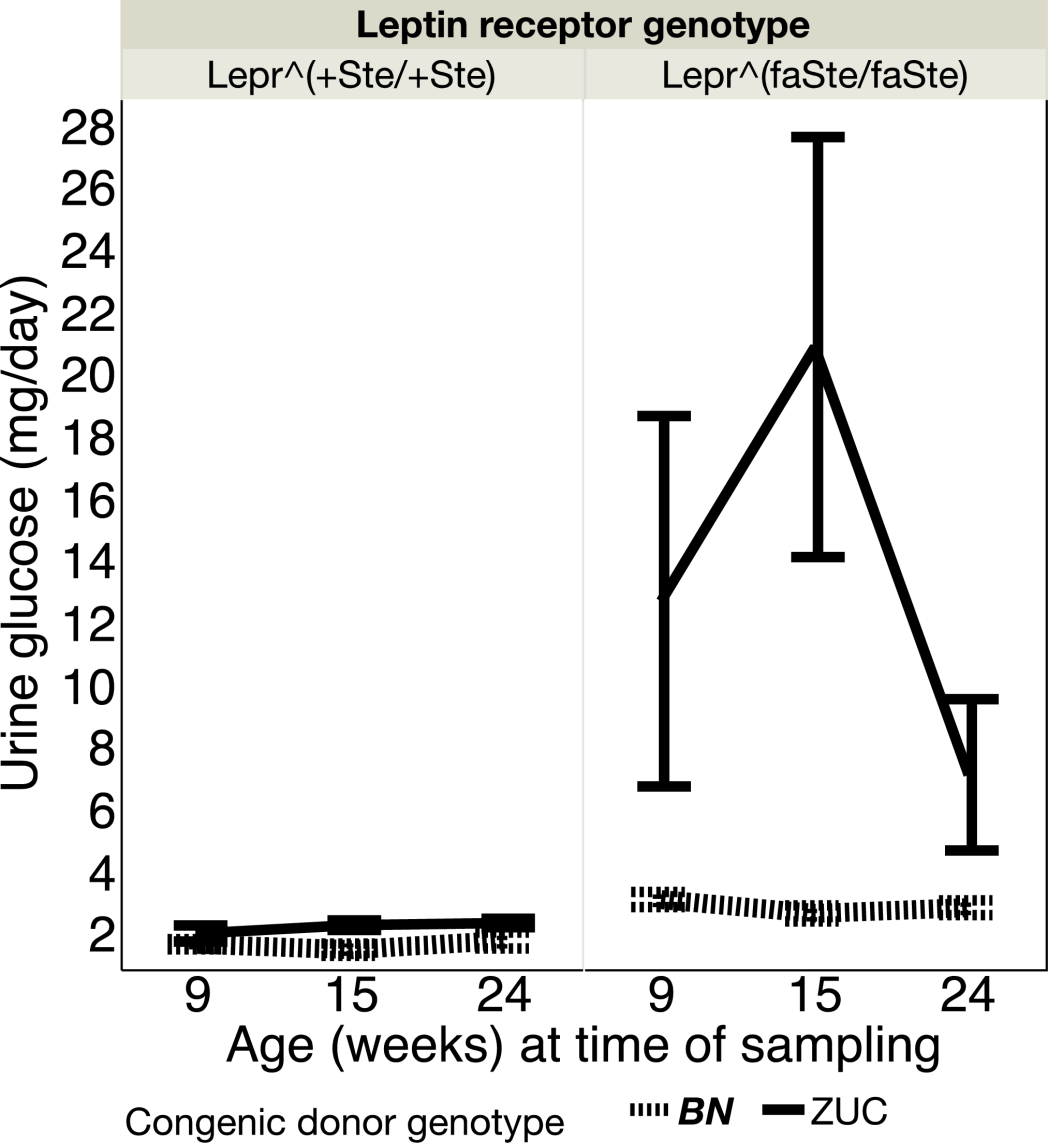
Urine glucose (mean ± SE, mg/d) at 9, 15 and 24 weeks of age is shown. Number of rats in each group: *Lepr*^*+Ste/+Ste*^, BN congenic N = 8, ZUC N = 6; *Lepr*^*faSte/faSte*^, BN congenic donor genotype N = 10, ZUC congenic donor genotype N = 5.

Urine glucose measured at 9, 15, and 24 weeks of age in female *Lepr* lean and *Lepr* fatty for congenic genotype BN and ZUC animals is shown in Fig 4.

*Lepr*^*faSte/faSte*^ females had higher urine glucose lost per day (mg/day) than *Lepr*^*+Ste/+Ste*^ females (P = 0.0023) and the BN congenic region resulted in lower urine glucose loss in both *Lepr* genotypes (P = 0.0113). There were significant interactions between age and *Lepr* genotype (P = 0.0081) and the congenic region genotype (P = 0.0063).

**Fig 5:**
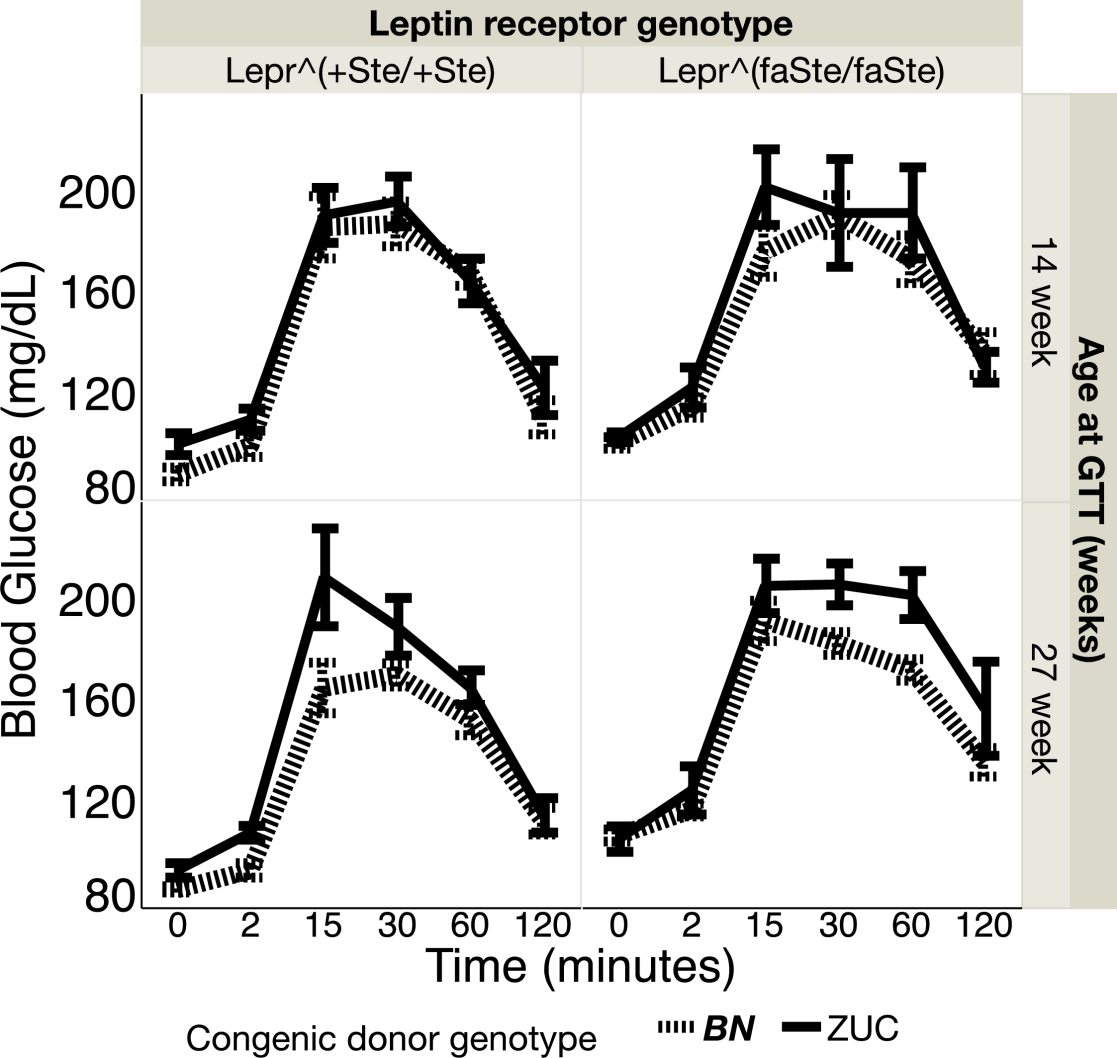
Blood glucose (mean ± SE, mg/dL) during GTT was measured as described in methods. Measurements were taken at 14 and 27 weeks of age in female *Lepr* lean and *Lepr* fatty for congenic genotype BN and ZUC animals. Number of rats in each group are the same for both ages: *Lepr*^*+Ste/+Ste*^, BN congenic N = 8, ZUC N = 6; *Lepr*^*faSte/faSte*^, BN congenic N = 10, ZUC N = 5.

Glucose tolerance test (GTT). At 14 weeks of age average fasting blood glucose (mean ± SE, mg/dL) at time zero before glucose injection in fatty *Lepr*^*faSte/faSte*^ female BN congenic donor genotype strain animals was 102 ± 2 (N = 10) and in fatty ZUC congenic donor genotype strain was 104 ± 2 (N = 5). At 27 weeks the values for fasting blood glucose in female *Lepr*^*faSte/faSte*^ BN congenic donor genotype strain animals was 108 ± 2 (N = 10) and for ZUC 107 ± 5 (N = 5). At 14 weeks of age average fasting blood glucose at time zero before glucose injection in lean *Lepr*^*+Ste/+Ste*^ female BN congenic donor genotype strain animals was 90 ± 3 (N = 8) and in lean ZUC congenic donor genotype strain was 102 ± 4 (N = 6). At 27 weeks the values for fasting blood glucose in *Lepr*^*+Ste/+Ste*^ BN congenic donor genotype strain animals was 87 ± 1 (N = 8) and for ZUC 95 ± 3 (N = 6). There are no significant BN vs. ZUC congenic donor genotype effects on fasting blood glucose but on average all the *Lepr*^*faSte/faSte*^ groups have prediabetic levels of fasting blood glucose (fasting blood glucose >100 mg/dL), and all the lean *Lepr*^*+Ste/+Ste*^ are normoglycemic. Blood glucose levels 2 hours after the glucose infusion during the 14 week GTT were 136 ± 8 and 132 ± 6 for fatty *Lepr*^*faSte/faSte*^ BN genotype congenic and ZUC genotype congenics, respectively. During the 27 week GTT blood glucose levels 2 hours after glucose were 137 ± 6 and 158 ± 19 for fatty *Lepr*^*faSte/faSte*^ BN genotype congenic and ZUC genotype congenics, respectively. Values for blood glucose during the GTT are shown in Fig 5.

Body weights (mean ± SE, g) at 14 weeks were: *Lepr*^*faSte/faSte*^ congenic donor genotype BN 345 ± 7 (N = 10) and congenic donor genotype ZUC strain 372 ± 11 (N = 5); for *Lepr*^*+Ste/+Ste*^ congenic donor genotype BN 198 ± 4 (N = 8) and congenic donor genotype ZUC strain 219 ± 2 (N = 6). At 27 weeks body weights were: *Lepr*^*faSte/faSte*^ congenic donor genotype BN 445 ± 11 (N = 10) and congenic donor genotype ZUC strain 480 ± 14 (N = 5); for *Lepr*^*+Ste/+Ste*^ congenic donor genotype BN 230 ± 3 (N = 8) and congenic donor genotype ZUC strain 253 ± 4 (N = 6).

At 14 weeks repeated measures ANOVA of blood glucose during GTT for fixed effects of congenic donor genotype, *Lepr* genotype, and time after injection revealed no statistically significant effects of congenic genotype, *Lepr* genotype, and congenic donor x *Lepr* genotype interaction. There was a highly significant effect of time after injection (p<0.0001). At 27 weeks repeated measures ANOVA of blood glucose during GTT for fixed effects of congenic donor genotype, *Lepr* genotype, and time after injection revealed no statistically significant effects of congenic genotype and congenic donor x *Lepr* genotype interaction. There was a highly significant effect of time after injection (p<0.0001) and only a suggestive effect of *Lepr* genotype (p = 0.0264).

Table 4 shows area under the curve data for blood glucose from GTT for lean and fatty females with ZUC and BN congenic donor chromosome genotypes. There is a significant effect of BN genotype (p = 0.0064) at 27 weeks of age.

**Table 4:** Glucose area under the curve (AUC) in females during GTT at 14 and 27 weeks of age.

*Lepr* genotype	*Lepr*^*+Ste/+Ste*^		*Lepr*^*faSte/faSte*^	
Congenic genotype	BN	ZUC	BN	ZUC
Number	8	6	10	N = 5, 14 wk. N = 4, 27 wk.
AUC 14 weeks	7929 ± 580^A^	7047 ± 705^A^	7493 ± 598^A^	8322 ± 1268^A^
AUC 27 weeks	6884 ± 287^B^	7778 ± 757^AB^	6769 ± 412^AB^	9134 ± 786^A^
Two-way ANOVA				
	Effect of *Lepr* genotype	Effect of BN Congenic genotype		*Lepr* x BN Congenic interaction
AUC 14 weeks	0.5865	0.9732		0.2721
AUC 27 weeks	0.2666	0.0064		0.1899

Data are mean ± SE. AUC was calculated as described in methods. Data analysis is by two-way ANOVA with mean differences calculated by Tukey HSD: means not sharing a superscript (^A, B^) are significantly different at p = 0.05.

Insulin tolerance test (ITT). At 13 weeks of age average fasting blood glucose (mean ± SE, mg/dL) at time zero before injection of insulin in fatty *Lepr*^*faSte/faSte*^ female BN congenic donor genotype strain animals was 108 ± 2 (N = 10) and in fatty ZUC congenic donor genotype strain was 101 ± 4 (N = 5 (n = 13). At 26 weeks the values for fasting blood glucose in female fatty *Lepr*^*faSte/faSte*^ BN congenic strain animals was 102 ± 3 (N = 10) and for ZUC strain 103 ± 3 (N = 5). At 13 weeks of age average fasting blood glucose at time zero before injection of insulin in *Lepr*^*+Ste/+Ste*^ female BN congenic donor genotype strain animals was 98 ± 2 (N = 8) and in lean ZUC congenic donor genotype strain was 102 ± 4 (N = 6). At 26 weeks the values for fasting blood glucose in female *Lepr*^*+Ste/+Ste*^ BN congenic donor genotype strain animals was 88 ± 2 (N = 8) and for ZUC strain 102 ± 4 (N = 6). There are no significant congenic donor BN vs. ZUC genotype effects and no significant age effects, but on average both groups of *Lepr*^*faSte/faSte*^ are prediabetic at 13 and 27 weeks (fasting blood glucose >100 mg/dl).

Body weights (mean ± SE, g) at 13 weeks for *Lepr* fatty animals were: BN congenic donor genotype 341 ± 6 (N = 10) and ZUC congenic donor strain 370 ± 10 (N = 5). At 26 weeks body weights for *Lepr*^*faSte/faSte*^ were: BN congenic 450 ± 11 (N = 10) and ZUC congenic donor genotype strain 485 ± 14 (N = 5). Body weights at 13 weeks for *Lepr* lean *Lepr*^*+Ste/+Ste*^ animals were: BN congenic donor genotype 202 ± 4 (N = 8), and ZUC congenic donor strain 224 ± 2 (N = 6). At 26 weeks body weights for *Lepr*^*+Ste/+Ste*^ were: BN congenic 236 ± 3 (N = 8) and ZUC congenic donor genotype strain 260 ± 4 (N = 6).

**Fig 6:**
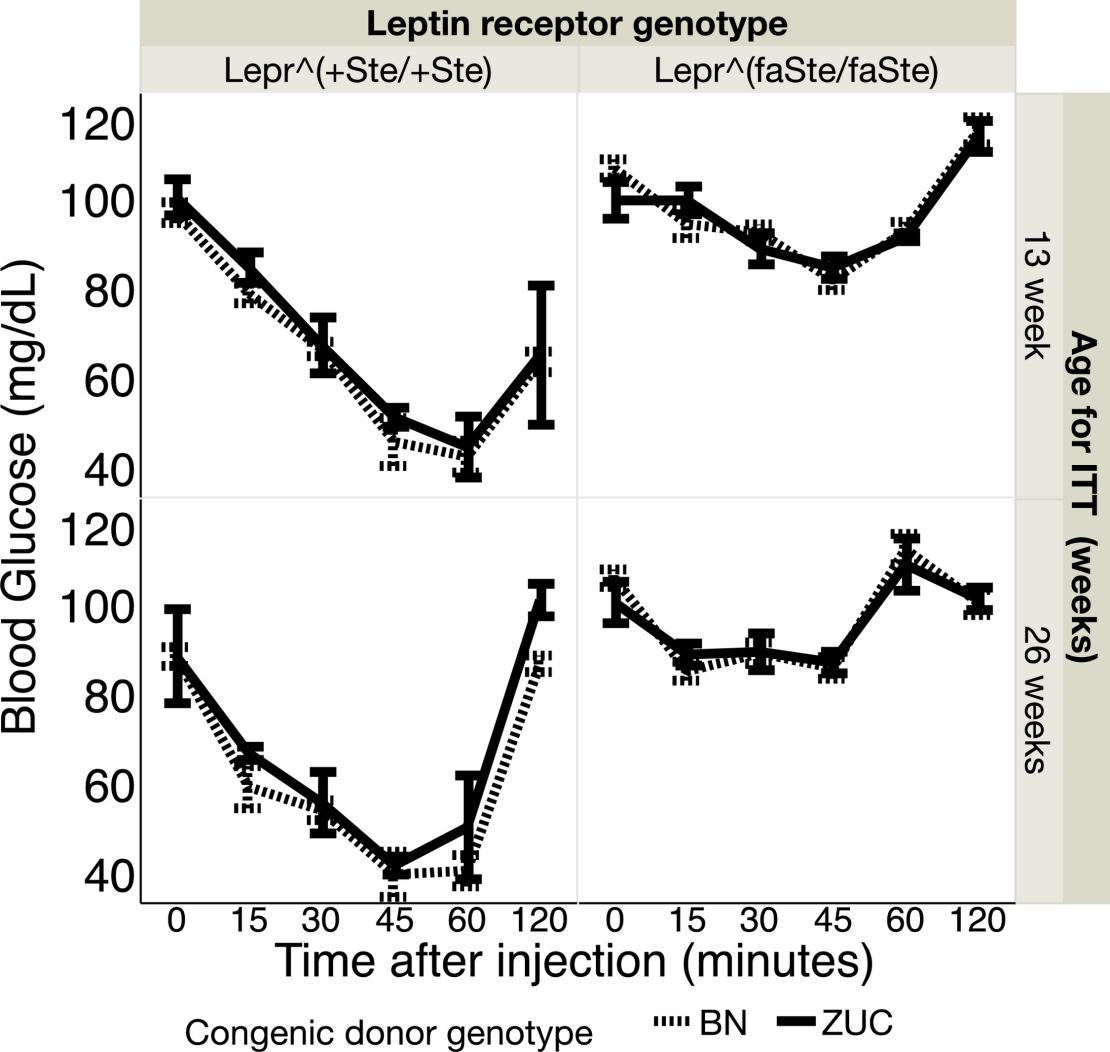
Glucose (mean ± SE, mg/dL) during ITT at 13 and 26 weeks of age in *Lepr* lean and *Lepr* fatty rats with congenic donor chromosome genotypes BN and ZUC. Number of rats in each group: *Lepr*^*+Ste/+Ste*^, 13 weeks BN congenic N = 8 at 0, 15, 30,60 min, N = 7 at 45 min and N = 3 at 120 min, 26 weeks BN congenic N = 8 at 0,15,30,120 min, N = 5 at 45 min and N = 4 at 60 min, ZUC N = 5 at 0, 15, 30, 45 min and N = 4 at 60 and 90 min13; *Lepr*^*faSte/faSte*^, 13 and 26 weeks BN congenic N = 10 all time points, ZUC N = 5 at 0,15,30,45, 90 min and N = 4 at 60 and 120 min.

Values for blood glucose during the ITT are shown in Fig 6. Repeated measures ANOVA for blood glucose during ITT revealed no statistically significant effects of congenic donor genotype or congenic donor genotype x time after insulin injection interaction. There are significant time (p<0.0001) and *Lepr* x time interaction (p<0.0001) effects at both 13 and 26 weeks of age. In addition there is a highly significant *Lepr* genotype effect (p = <0.0002).

### Quantitative PCR of six positional candidate genes

Differences in gene expression between congenic and background strains can be correlated with susceptibility to disease. We performed quantitative PCR (qPCR) on six positional candidate genes that are located in the congenic donor region and discussed in the introduction to determine if there are significant chromosome 1 congenic donor region genotype effects. We performed qPCR on samples taken from the whole kidney, and from kidney cortex and medulla, dissected by visual inspection. Groups examined included *Lepr* lean and *Lepr* obese and BN congenic and ZUC congenic donor chromosome genotype females at 15 weeks. Values were normalized to Tata-box binding protein expression as the control gene. Fold-expression was then calculated by setting lean ZUC genotype animals to an average of 1.0-fold. We tested all groups for kidney region effects and congenic and *Lepr* effects (Table 5). All six genes examined have highly significant chromosome 1 BN congenic genotype effects and kidney part effects. Three genes *Umod*, *Rab11-fip2*, and *Sorcs2*, had significant *Lepr* genotype effects, but *Scnn1b*, *Pdilt*, and *Pkd2l1* did not have significant effects from *Lepr* genotype. *Sorcs2* exhibited significant effects for congenic and *Lepr* genotype and all interactions.

**Table 5:** Kidney gene expression in female Zucker rats comparing Zucker and congenic genotypes at 15 weeks of age.

Zucker genotype	Congenic genotype	No.	Kidney part	*Umod* fold	*Rab11-* *Fip2* fold	*Sorcs1* fold	*Scnn1b* fold	*Pdilt* fold	*Pkd2l1* fold
*Lepr*^*+Ste/+Ste*^	BN	10	Cortex	1.57 ± 0.10^CD^	1.49 ± 0.26^ABC^	0.156 ± 0.007^B^	0.579 ± 0.028^B^	0.708 ± 0.155^B^	0.892 ± 0.013^C^
			Medulla	0.407 ± 0.059^D^	0.705 ± 0.100^CDE^	22.7 ± 4.4^B^	0.084 ± 0.013^B^	No data	8.83 ± 1.83^AB^
			Whole	2.43 ± 0.14^CD^	0.873 ± 0.157^BCDE^	173.8 ± 34.8^A^*	0.069 ± 0.003^B^	0.114 ± 0.003^B^	0.577 ± 0.018^C^
	ZUC	4	Cortex	1.26 ± 0.35^CD^	2.47 ± 1.08^A^	1.31 ± 0.53^B^	3.79 ± 2.15^A^	6.58 ± 3.74^A^	0.978 ± 0.099^BC^
			Medulla	1.63 ± 0.70^CD^	0.962 ± 0.235^ABCDE^	0.960 ± 0.09^B^	1.09 ± 0.16^B^	No data	5.21 ± 4.46^ABC^
			Whole	2.32 ± 0.83^CD^	2.00 ± 0.59^AB^	1.30 ± 0.23^B^	1.34 ± 0.46^B^	1.38 ± 0.36^B^	1.42 ± 0.50^BC^
*Lepr*^*faSte/faSte*^	BN	11	Cortex	6.10 ± 0.41^B^	0.441 ± 0.034^DE^	0.169 ± 0.006^B^	0.949 ± 0.102^B^	0.563 ± 0.110^B^	1.12 ± 0.04^C^
			Medulla	2.19 ± 0.16^CD^	0.124 ± 0.012^DE^	12.8 ± 2.05^B^	0.178 ± 0.011^B^	No data	13.0 ± 2.1^A^
			Whole	11.4 ± 0.7^A^	0.060 ± 0.01^E^	3.85 ± 1.10^B^	0.208 ± 0.011^B^	0.185 ± 0.017^B^	13.8 ± 2.6^A^
	ZUC	9	Cortex	2.72 ± 0.22^CD^	1.03 ± 0.26^BCD^	0.474 ± 0.151^B^	5.52 ± 0.61^A^	1.42 ±0.37^B^	0.933 ± 0.029^C^
			Medulla	1.14 ± 0.20^D^	0.304 ± 0.063^DE^	1.81 ± 0.41^B^	0.268 ± 0.026^B^	No data	0.580 ± 0.134^C^
			Whole	3.89 ± 1.11^BC^	0.613 ± 0.226^CDE^	3.50 ± 1.28^B^	0.374 ± 0.038^B^	0.658 ± 0.199^B^	0.513 ± 0.119^C^
Three-way ANOVA									
Zucker *Lepr* genotype				**<0.0001**	**<0.0001**	**<0.0001**	0.7731	**0.0021**	0.0383
Congenic BN/ZUC genotype				**<0.0001**	**<0.0001**	**<0.0001**	**<0.0001**	**<0.0001**	**<0.0001**
Kidney part gene expression				**<0.0001**	**<0.0001**	**<0.0001**	**<0.0001**	**0.0004**	**<0.0001**
Zucker *Lepr* genotype x Congenic genotype				**<0.0001**	0.2127	**<0.0001**	0.5054	**0.0026**	**0.0001**
Kidney part x Zucker genotype				**<0.0001**	0.1599	**<0.0001**	0.0130	0.0144	0.0106
Kidney part x Congenic genotype				**<0.0001**	0.1325	**<0.0001**	**<0.0001**	0.0090	**0.0025**
Kidney part x Zucker x Congenic				**0.0047**	0.7611	**<0.0001**	0.0538	0.0257	0.0138

Data are mean ± SE. Mean differences calculated by Tukey HSD: means not sharing a superscript (^A, B, C, D, E^) are significantly different at p = 0.05. P values in bold are significant at p = 0.05 when corrected for multiple testing using the FDR.

*N = 9.

Zucker genotype compares two groups: lean *Lepr*^*+Ste/+Ste*^ versus *Lepr*^*faSte/faSte*^. Congenic BN genotype compares two genotypes: homozygous BN for *D1Rat183*-*D1Rat90* versus homozygous ZUC for the entire genome. Kidney part compares three samples: cortex, medulla and whole kidney.

## Conclusions

Our results show that female fatty UC Davis ZUC strain animals have a mild prediabetes phenotype with fasting blood glucose above 100 mg/dL, when using American Diabetes Association (ADA) criteria for prediabetes in humans of >100 mg/dL for fasting plasma glucose [54]. The results for fatty animals assessed at 27 weeks of age also meet ADA criteria for prediabetes in humans of >140 mg/dL 2 hours after an oral glucose tolerance test [54]. Because the fasting blood glucose measurements were made using an uncalibrated OneTouch Ultra Meter, then it is possible that actual glucose levels are less than 100 mg/dL. We conclude that UC Davis ZUC strain animals either have prediabetic blood glucose levels or they almost do when using ADA criteria for humans. The congenic captures BN strain alleles that reduce blood glucose area under the curve (AUC) levels compared with congenic donor genotype ZUC animals during a glucose tolerance test (Table 4) and that also reduce urine glucose lost per day (Fig 4). Since there were no effects of the BN congenic donor region on fasted glucose or insulin tolerance tests, then the results suggest that the BN chromosome 1 donor region reduces the mild glucose intolerance observed during GTT tests of the UC Davis female ZUC strain animals, but there appears to be no congenic donor region genotype effect on insulin resistance. However, there is a clear effect of *Lepr* genotype on insulin resistance in females, with fatty animals being insulin resistant (Fig 6). We have not measured any traits in ZUC from other vendors or institutions, so we do not know how our results could compare to other ZUC strains. Thus, the BN.ZUC-*D1Rat183*-*D1Rt90* congenic strain is a model for glucose intolerant type 2 diabetes with similar effects in both males and females, although the type 2 diabetes is more severe in males and is very mild in females. The results are consistent with the hypothesis that both genders can be used to map the underlying genes that influence phenotypes of type 2 diabetes and kidney disease. Finally, whether or not the fatty *Lepr* deficient animals are labeled as prediabetic, the BN donor region of the congenic improves glucose tolerance, i.e., its presence results in reduced blood glucose levels during a glucose tolerance test.

The food intake data shows that BN genotype congenic strain females eat significantly less than ZUC strain animals, particularly at older ages. Previous data from our lab has shown that food intake restriction of ZUC strain animals is correlated with reduced urinary albumin excretion and reduced plasma triglyceride levels [17, 18]. Thus, our hypothesis is that the reduced food intake of the BN genotype strain congenic (Fig 1) may contribute to their reduced plasma triglyceride levels (Table 3) [18]. In a previous publication we demonstrated that male fatty ZUC genotype animals had much higher urine volumes, particularly after 15 weeks of age [14]. We have observed significant effects of ZUC congenic donor genotype to increase urine volume of female *Lepr*^*faSte/faSte*^ animals (Fig 2). Thus, males and females show similar effects of the congenic donor region genotype on urine volume. There were no significant chromosome 1 congenic region genotype effects for ACR in UC Davis females (Fig 3). In males the chromosome 1 congenic region genotype had a significant effect on ACR, so males and females have different phenotypes for this trait in our colony [14].

Other investigators have examined kidney function in male and female ZUC. In a study of lean and fatty Zucker males and fatty female Zucker rats observed at 16 weeks, obese females have more than 15-fold higher ACR than lean males and about half the ACR of fatty males [55]. At 15 weeks we observed that ACR (mg UAE per mg creatinine) was 2.6 mg/mg in fatty females and 6.4 mg/mg in fatty males. In comparison, Dominguez et al [55] reported 1.2 mg urine albumin/mg creatinine for males and 0.67 mg/mg for females. Thus, females reproducibly develop a milder kidney disease than males.

We did not assess kidney pathology in females in the present study. A cohort study of 177,570 individuals conducted over an almost 10 year span searched for risk factors for end stage renal disease (ESRD) [5]. This study reported that the two most potent risk factors were proteinuria (hazard ratio of 7.9 for 3 to 4+ on urine dipstick) and excess weight (hazard ratio 4.39 for class II or III obesity). We have previously shown that in the UC Davis Zucker fatty male model ACR is increased 42-fold from 9 to 28 weeks of age with the ZUC background strain genotype on chromosome 1 [14, 15]. For females ACR in fatty animals with the chromosome 1 ZUC genotype also increased 16-fold over the same timespan, consistent with mild kidney disease.

Although female ZUC are often considered to be non-diabetic, our GTT results show that several groups (fasted blood glucose and blood glucose 2 hours after start of the GTT, Fig 5) have average blood glucose levels in the prediabetic range [54]. This result may be because we are using a unique colony of ZUC that has been maintained continuously at UC Davis since 1982, or because of some other local peculiarity, such as diet, microbiome or animal care. We have not compared the UC Davis ZUC colony to ZUC strain animals from vendors or other institutions. There were no statistically significant congenic donor genotype or congenic donor genotype x time after injection effects for repeated measures ANOVA of ITT at 13 and 26 weeks of age. Although the present paper does not show results for males because they were previously published [14], GTT and ITT analysis of males revealed essentially the same congenic genotype x time after injection effects on repeated measures ANOVA of the BN donor region on fasting blood glucose after GTT.

Do our results identify genes responsible for kidney disease? Significant congenic genotype effects on gene expression (Table 5) means that BN alleles of these genes result in significantly different mRNA levels than ZUC alleles of these genes. We do not know if these expression differences are due to cis or trans effects, because all the animals used have only one of two genotypes–completely ZUC or homozygous for the congenic donor region BN.ZUC-*D1Rat183*-*D1Rat90*. Since we did not independently validate dissection using qPCR for genes known to be specific to cortex or medulla, our results generate hypotheses about levels of gene expression in cortex and medulla, but we cannot be definitive about location of expression in the kidney. Despite this limitation, we observe highly significant differences in gene expression by kidney part for all six genes quantitated, consistent with the hypothesis that we have separated parts of the kidney with different levels of expression for these six genes. This also suggests that all six remain positional candidates to influence other phenotypes that map to the BN congenic donor region in females, such as urine volume, urine glucose and glucose response to a GTT.

Urine volume is influenced by many factors, including disease, drugs, and behaviors that affect what and how much is drunk. Many papers have identified positive correlations between type 2 diabetes and urine volume [56, 57]. What is not clear is if type 2 diabetes is always causal for increases in urine volume, if increased urine glucose is always causal for increased urine volume, or if the correlation means that type 2 diabetes or elevated urine glucose increases the risk for increased urine volume. Our own data show that 24 week old female fatty ZUC congenic donor genotype animals have higher urine volume than 15 week female fatty ZUC congenic donor genotype animals, but urine glucose lost per day is less at 24 weeks of age than at 15 (Figs 2 and 4). Measurements of urine volume and urine glucose were contemporaneously determined in the same animals so we have directly quantitated the relationships by regression analysis. We observed no significant correlations of urine volume and urine glucose concentration (mg/dL) when including all 4 groups (*Lepr* and congenic donor genotypes), with each time point (9, 15 and 24 week) analyzed separately. We also observed no significant correlations of urine volume and urine glucose concentration (mg/dL) when analyzing only fatty *Lepr*^*faSte/faSte*^ and both congenic donor genotypes–BN and ZUC. We also observed no significant correlations when analyzing only fatty *Lepr*^*faSte/faSte*^ animals with the ZUC genotype for the congenic donor region. Thus, our results are not consistent with the hypothesis that urine glucose concentration is the only determinant of urine volume in the UC Davis Zucker rat model. However, as Fig 2 shows there is a strong congenic donor region chromosome 1 genotype effect on urine volume, but the physiological pathway affected is unknown. Four genes in Table 5 (*Umod*, *Sorcs1*, *Pdilt* and *Pkd2l1*) have highly significant *Lepr* genotype x congenic donor genotype interaction effects that are consistent with their having a role in controlling urine volume, but our data do not prove causality for any of these. Our results also do not exclude other genes in the congenic donor region that were not tested in Table 5. Thus, the specific gene(s) responsible for the congenic donor genotype effect on urine volume in fatty *Lepr*^*faSte/faSte*^ rats is/are unknown.

We have shown that the UC Davis ZUC female model has mild prediabetes levels of glucose in fasted blood, 2 hours after start of a glucose tolerance test, and mild glucose intolerance during a glucose tolerance test. The *Lepr* fatty UC Davis female congenic donor genotype ZUC strain animal has greatly increased plasma triglyceride, increased urine volume compared with *Lepr* lean controls and loses much more glucose in urine. Several of these traits are reduced in a congenic with a BN donor region on chromosome 1 from microsatellite markers *D1Rat183*-*D1Rat90*.

## Abbreviations

BAT: Brown Adipose Tissue
MWAT: Mesenteric white adipose tissue
ACR: Urinary albumin to creatinine ratio (mg/mg)
*Lepr*: *leptin receptor*
T2D: type 2 diabetes
ESRD: end stage renal disease
BMI: body mass index
UAE: urinary albumin excretion
QTL: quantitative trait locus
eGFR: estimated glomerular filtration rate
GTT: glucose tolerance test
ITT: insulin tolerance test
TG: triglyceride
ALT: alanine aminotransferase
*Tbp*: TATA-binding protein
ANOVA: analysis of variance
